# New species of *Prosopodesmus* Silvestri, 1910 (Diplopoda, Polydesmida, Haplodesmidae) from Queensland, Australia

**DOI:** 10.3897/zookeys.190.3276

**Published:** 2012-05-04

**Authors:** Robert Mesibov

**Affiliations:** 1 Queen Victoria Museum and Art Gallery, Launceston, Tasmania 7250, Australia

**Keywords:** Diplopoda, Polydesmida, Haplodesmidae, millipede, Australia, Queensland

## Abstract

*Prosopodesmus crater* **sp. n.**, *Prosopodesmus kirrama* **sp. n.** and *Prosopodesmus monteithi* **sp. n.** are described from the Wet Tropics of north Queensland. The hothouse species *Prosopodesmus panporus* Blower & Rundle, 1980 is recorded from rainforest on Queensland’s Cape York Peninsula, where it is likely to be native.

## Introduction

*Prosopodesmus* Silvestri, 1910 is a small genus of tropical haplodesmid Polydesmida with three transverse rows of large tubercles on the metatergites and downward-bending paranota (Golovatch et al. 2009). The unbranched, sickle-shaped gonopod telopodites normally lie parallel to the main body axis in a hollow formed by ventromedial concavities on the gonocoxae.

Two species have been described in *Prosopodesmus*. The type species *Prosopodesmus jacobsoni* Silvestri, 1910 is a pantropical tramp first collected at Jakarta (‘Batavia’) on Java. It has since been found in Florida and Puerto Rico (USA), Haiti (as the synonymous *Homodesmus parvus* Chamberlin, 1918), St Eustatius (Leeward Islands, West Indies), Panama, Brazil, Zanzibar, India, New Caledonia and the Galapagos Islands (Hoffman 1999). It has recently been confirmed as present on Taiwan (Golovatch et al. 2011) and there are *Prosopodesmus jacobsoni* records from Louisiana (USA) (Shelley and Golovatch 2000), Christmas Island (Indian Ocean) (Jeekel 2006) and Fiji (Akkari and Enghoff 2011). The native range of *Prosopodesmus jacobsoni* is unknown.

*Prosopodesmus panporus* Blower & Rundle, 1980 was described from tropical plant hothouses at the Royal Botanic Gardens at Kew, England (Blower and Rundle 1980). The describers did not suggest a native range for the species: “Presumably *Prosopodesmus panporus* lurks in some unworked tropical habitat.” (Blower and Rundle 1980, p. 32).

Two more species were added to *Prosopodesmus* when the Japanese genus *Rhipidopeltis* Miyosi, 1958 was made a junior synonym (Golovatch et al. 2009, Golovatch et al. 2010). *Prosopodesmus sinuatus* (Miyosi, 1958) is known only from far southern Honshu and *Prosopodesmus similis* (Haga, 1968) from Kyushu, to the south of Honshu across the Kanmon Strait.

While sorting museum samples of Pyrgodesmidae from eastern Australia, I found four species of pyrgodesmid-like *Prosopodesmus* in Berlese samples from rainforest in tropical north Queensland. Three of the species are new, while the fourth is *Prosopodesmus panporus*.

## Methods

‘Male’ and ‘female’ in the text refer to adult individuals. All specimens are stored in 75–80% ethanol in their respective repositories.

Whole, selected specimens and rings 7 of selected males were cleared in 80% lactic acid and temporarily mounted in 60% lactic acid for optical microscopy. Preliminary gonopod drawings were traced from digital images acquired with an eyepiece video camera temporarily fitted to an optical microscope. Body parts of uncleared specimens were temporarily mounted in a 1:1 glycerol:water mixture for examination and measurement. Photomicrographs were taken with a Canon EOS 1000D digital SLR camera mounted on a Nikon SMZ800 binocular dissecting microscope equipped with a beam splitter. Specimens for scanning electron microscopy were briefly air-dried, temporarily fixed to a stub with double-sided adhesive tape or a sticky carbon pad, examined uncoated with a FEI Quanta 600 operated in low-vacuum mode, then returned to alcohol. Images and drawings were prepared for publication using GIMP 2.6. The map figure was generated using ArcView GIS 3.2.

The specimen localities given below are also listed in Appendix 2 in table form. Latitude and longitude (WGS84 datum) are from museum collection databases. The uncertainty for each locality (in square brackets) is the radius of a circle around the stated position, and is my own estimate.

Abbreviations: ANIC = Australian National Insect Collection, Canberra, Australian Capital Territory; IEA = Instituto di Entomologia Agraria, Portici, Italy; NHM = Natural History Museum, London, UK; Qld = Queensland; QM = Queensland Museum, Brisbane, Qld; VMNH = Virginia Museum of Natural History, Martinsville, Virginia, USA.

## Results

### Order Polydesmida Pocock, 1887. Suborder Polydesmidea Pocock, 1887. Family Haplodesmidae Cook, 1895

#### *Prosopodesmus* Silvestri, 1910

*Prosopodesmus* Silvestri 1910: 360. Attems 1914: 177, 1926-1930: 136, 1940: 292. Brölemann 1916: 570, 1920: 225. Jeekel 1971: 348. Blower and Rundle 1980: 27. Hoffman 1980: 174, 1999: 432. Golovatch et al. 2009: 2, 43. Golovatch et al. 2010: 33.

**Type species.** *Prosopodesmus jacobsoni* Silvestri, 1910, by original designation.

**Other included species.** *Prosopodesmus crater* sp. n., *Prosopodesmus kirrama* sp. n., *Prosopodesmus monteithi* sp. n., *Prosopodesmus panporus* Blower and Rundle, 1980, *Prosopodesmus similis* (Haga, 1968), *Prosopodesmus sinuatus* (Miyosi, 1958).

*Homodesmus* Chamberlin 1918: 222. Attems 1926-1930: 141, 1940: 294. Loomis 1950: 166 (synonymised with *Prosopodesmus*). Jeekel 1971: 331. Hoffman 1980: 174, 1999: 432. Golovatch et al. 2009: 40, 43.

**Type species.** *Homodesmus parvus* Chamberlin, 1918. (Synonymised with *Prosopodesmus jacobsoni* in Loomis 1950: 166)

*Rhipidopeltis* Miyosi 1958: 297. Hoffman 1980: 174. Shelley et al. 2000: 127. Golovatch et al. 2009: 2, 43 (synonymised with *Prosopodesmus*). Golovatch et al. 2010: 33.

**Type species.** *Rhipidopeltis sinuata* (recte *sinuatus*) Miyosi, 1958.

#### Prosopodesmus crater sp. n.

urn:lsid:zoobank.org:act:D20FAE64-B7BC-4A68-9733-4EB33AC6CAAF

http://species-id.net/wiki/Prosopodesmus_crater

Figs 1A 2A 3A 5A

##### Holotype.

Male, Eacham National Park, Qld, 17°18'S, 145°37'E [±2 km], 760 m, 28 June 1971, R.W. Taylor and J. Feehan, berlesate 344, rainforest, ANIC 64-000213.

##### Paratypes.

1 male, 2 stadium 7 males, 2 stadium 7 females, 1 stadium 6 male, details as for holotype, ANIC 64–000212; 1 male, details as for holotype but 1–7 October 1972, R.W. Taylor, berlesate 428, ANIC 64–000214; 1 male, 2 females, Cathedral Fig, Qld, 17°10'52"S, 145°39'26"E [±500 m], 720 m, 7 February 1996, G. Monteith, berlesate 907, rainforest, sieved litter, QM S37593; 1 male, 1 female, Downey Creek, 25 km SE of Millaa Millaa, Qld, 17°40'48"S, 145°46'58"E [±500 m], 400 m, 7 December 1988, G. Monteith and G. Thompson, berlesate 813, rainforest, sieved litter, QM S91625.

**Other material.** 1 male, Cammoo Caves near Rockhampton, Qld, 23°10'S, 150°28'E [±2 km], 25 October 1976, R.W. Taylor and T.A. Weir, berlesate 535, dense, low, closed forest, ANIC 64–000215.

**Figure 1.**
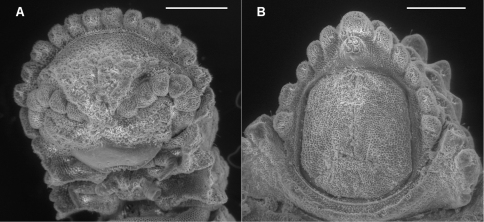
**A** Ventral view of head of *Prosopodesmus crater* sp. n., paratype, ANIC 64–000212, showing 12 lobes on anterior edge of collum, antennae retracted below edges of collum and ring 2 tergite, and textured frons with smooth clypeus. **B** Ventral view of telson of *Prosopodesmus monteithi* sp. n., QM S91632, showing 5+5 lobe pattern on edge of preanal ring and apical epiproct.

**Figure 2.**
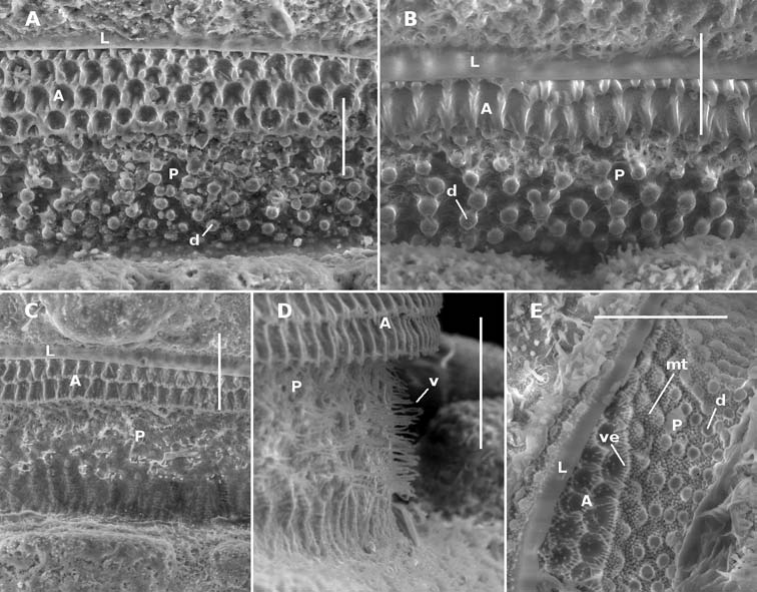
Midbody limbus and prozonite of *Prosopodesmus* species, dorsal views. **A** *Prosopodesmus crater* sp. n., paratype, QM S37593. **B** *Prosopodesmus kirrama* sp. n., paratype, QM S91627. **C, D** *Prosopodesmus monteithi* sp. n., QM S91632. **E** *Prosopodesmus panporus* Blower and Rundle, 1980, ANIC 64–000118. **L** limbus **A** anterior portion of prozonite **P** posterior portion of prozonite **d** disk **mt** microtubercles **v** villi **ve** microvillose extensions. Scale bars: **A, B, E** = 0.05 mm, **C, D** = 0.1 mm.

**Figure 3.**
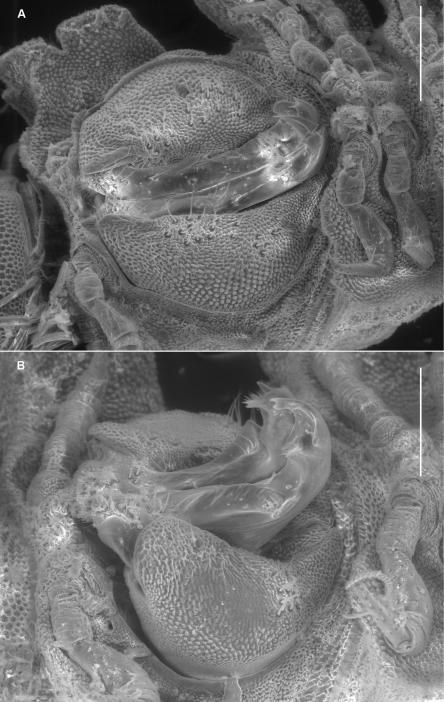
Left ventrolateral views of gonopods of *Prosopodesmus* species. **A** *Prosopodesmus crater* sp. n., paratype, ANIC 64–000212. **B** *Prosopodesmus kirrama* sp. n., paratype, QM S91627. Tip of left gonopod telopodite of *Prosopodesmus crater* is broken. Scale bars = 0.2 mm. Image contrast is low because specimens are uncoated.

##### Diagnosis.

Males and females with head + 20 rings; adults 7–8 mm long; midbody metatergites typically with 3 transverse rows of 10 large tubercles; posterior portion of prozonite with small disks, no microtubercles; ozopores not on porosteles; gonopod telopodite with single posteriad bend, near tip.

##### Description.

Male with head + 20 rings, ca 7 mm long. Colour in alcohol pale yellow; lightly encrusted with fine soil particles. Ring 12 with maximum vertical diameter 0.7 mm; maximum width (including paranota) 0.9 mm and 1.6X prozonite width; paranotal length ca 1/2 of total ring length.

Head (Fig. 1A) facing ground, covered by anterior collum edge in dorsal view; vertex microtuberculate; frons with large, irregular tubercles with microvillose texture; clypeus smooth, sparsely setose ventrally. Antennal sockets separated by slightly more than a socket diameter. Retracted antennae with distalmost antennomeres held in groove formed by lateral edge of frons anteriorly and confluent lateral collum and ring 2 tergite edges posteriorly. Antennomere relative widths (5,6)>(2,3,4), relative lengths 6>5>(2,3,4).

Collum with 12 lobes along anterior edge (Fig. 1A). Collum, ring 2 tergite and metatergites 5–15 about equally wide; rings 3,4 slightly narrower; rings16–18 progressively narrowing. Collum, tergites and metatergites textured with large, low tubercles, each with irregular, roughened, microvillose fine structure; 3 transverse rows of typically 10+10+10 tubercles on midbody metatergites. Dorsal and lateral setae on collum, tergites and metatergites sparse, bisegmented, the distal portion flattened, slightly flared at tip and minutely toothed along distal edge. Anterior portion of prozonite (Fig. 2A) cellular, posterior portion irregularly covered with small, more or less round, variably sized, convex disks. Limbus a smooth, straight-edged lamella.

Ring 2 tergite edge lower than collum edge and ring 3 tergite edge. Paranota set low on body and declined at about 45°, subquadrate in dorsal view; lateral margin notched into 3 lobes at ca 1/8 and 1/2 the paranotal length.

Ozopore not on porostele, inconspicuous near posterolateral corner of paranotum; pore formula 5, 7, 9, 10, 12, 13, 15–19.

Sternites as wide as long. Legs short, hidden by paranota in dorsal view. Relative podomere lengths (femur, tarsus)>prefemur>(postfemur, tibia).

Spiracles not evident.

Telson (as for *Prosopodesmus monteithi* sp. n in Fig. 1B) facing ground; preanal ring with 5 lobes on each side and 1 larger lobe (epiproct) apically. Spinnerets recessed in individual chambers, basal sheaths with unnotched distal edges; setal shafts smooth. Paraprocts more or less flat, rounded-rectangular; paraproctal setae close to and equidistant from margin. Hypoproct trapezoidal, hypoproctal setae not on raised tubercles.

Gonopore inconspicuous on leg 2 coxa. Ring 6 metatergite with slight medial excavation on posterior margin ventrally, accommodating tips of retracted gonopod telopodites. Aperture ovoid, as wide as ring 7 prozonite and extending anteriorly to occupy ventral portion of prozonite. Gonocoxae (Fig. 3A) massive, slightly tapering posteroventrally, surface densely microtuberculate; flat ventrally with a few setae near medial side; medial side slightly concave.

Telopodite base in shallow recess at posteroventral corner of gonocoxa. Telopodites (Figs 3A, 5A) slender, straight, more or less uniformly wide, parallel, just touching when retracted, slightly convex anteriorly, slightly concave ventrally; bent posteriorly at tip; with thin, rounded tabs projecting posteriorly from lateral edge at between 1/4 and 1/2 the telopodite length and just basal to the apical bend; with thin, rounded tab similarly extending the medial edge just distal to the bend. Telopodites with a few larger setae to about 1/2 telopodite length on posterior surface laterally; with numerous fine setae around and just inside basal concavity into which the prominent cannula inserts. Prostatic groove running straight to small, low mound on middle of posterior telopodite surface at about 3/4 telopodite length; mound covered with ‘hairpad’ of slender, pointed villi.

Female with head + 20 rings; a little larger than male, ca 8 mm long. Epigyne ca 1/3 ring width, slightly raised, rounded rectangular with straight distal edge; cyphopods not examined.

##### Distribution.

Rainforest on the Atherton Tableland southwest from Cairns, Queensland, with a known north-south range of ca 40 km (Fig. 8). There is also one questionable record from the Cammoo Caves area in central coastal Queensland. (See Remarks.)

##### Etymology.

Latin *crater*, ‘cup’. The type locality surrounds Lake Eacham, a crater lake on the Atherton Tableland in far north Queensland.

##### Remarks.

The male in ‘Other material’ is from a site nearly 800 km south and east from the nearest other *Prosopodesmus crater* locality. This specimen is from the same ANIC berlesate that indicated a similar 800+ km disjunction in the range of the unrelated millipede *Asphalidesmus magnus* Mesibov, 2011 (Mesibov 2011). Although the Cammoo Caves *Prosopodesmus crater* is very slightly different in gonopod details from those collected on the Atherton Tableland, the most likely explanation is that the Cammoo Caves specimens of *Prosopodesmus crater* and *Asphalidesmus magnus* were actually collected in the Wet Tropics, and that the locality labels are incorrect.

#### Prosopodesmus kirrama sp. n.

urn:lsid:zoobank.org:act:308DBF9D-BF94-4705-9F0B-15F44ECFD6BD

http://species-id.net/wiki/Prosopodesmus_kirrama

Figs 2B 3B 5B

##### Holotype.

Male, Douglas Creek Road, Kirrama Range, Qld, 18°13'30"S, 145°48'13"E [±500 m], 800 m, 10 December 1986, G. Monteith and G. Thompson, berlesate 731, rainforest, sieved litter, QM S91629.

##### Paratypes.

1 male, 2 stadium 7 males, details as for holotype, QM S91628; 2 males, 3 females, 1 stadium 6 male, near Yuccabine Creek, Kirrama Range, Qld, 18°12'21"S, 145°45'47"E [±500 m], 700 m, 10 December 1986, G. Monteith and G. Thompson, berlesate 732, QM S91626; 2 males, Kirrama Range, Qld, 18°12'57"S, 145°47'15"E [±500 m], 700 m, 9 December 1986, G. Monteith and G. Thompson, berlesate 730, QM S91627.

##### Other material.

1 female, Upper Broadwater valley, Cardwell Range, Qld, 18°19'15"S, 145°58'34"E [±500 m], 800 m, 16 January 1987, S. Hamlet, berlesate 759, rainforest, sieved litter, QM S91631; 3 stadium 7 females, 1 stadium 6 female, same details but 700 m, 20 December 1986, G. Monteith, G. Thompson and S. Hamlet, berlesate 745, QM S91630.

##### Diagnosis.

Males and females with head + 20 rings; adults 8–9 mm long; midbody metatergites typically with 3 transverse rows of 12 large tubercles; posterior portion of prozonite (Fig. 2B) with small disks, no microtubercles; ozopores not on porosteles; gonopod telopodite with two posterior bends, one at about midlength and one near tip, the more basal bend marked by strong anterior production of telopodite.

##### Description.

As for *Prosopodesmus crater*, differing in the following details:

Male/female lengths ca 8/9 mm, respectively. Ring 12 with maximum vertical diameter 0.8 mm; maximum width (including paranota) 1.2 mm and 1.7X prozonite width. Ring 2 slightly narrower than collum. 3 transverse rows of typically 12+12+12 tubercles on metatergites.

Gonocoxae (Fig. 3B) massive, strongly tapering anteroventrally and posteroventrally. Telopodite (Figs 3B, 5B) strongly produced anteriorly at about midlength, bending posteriorly there and again near tip. Hairpad mound medial on posterior surface midway between second bend in telopodite and tip. Thin, rounded tab on medial edge of telopodite just below level of hairpad; thin, rounded tabs on lateral edge at level of first bend and just distal to hairpad; narrow longitudinal ridge medially on posterior telopodite surface from near base to near first bend.

##### Distribution.

Rainforest in the mountains southwest from Tully and northwest from Ingham, Queensland, with a known north-south range of ca 25 km (Fig. 8).

##### Etymology.

For the type locality, the Kirrama Range.

#### Prosopodesmus monteithi sp. n.

urn:lsid:zoobank.org:act:1E427018-6159-407B-9178-C6AA835A723F

http://species-id.net/wiki/Prosopodesmus_monteithi

Figs 1B 2C, 2D 4 5C 6

##### Holotype.

Male, 2 km SE of Mt Spurgeon via Mt Carbine, Qld, 16°27'17"S, 145°12'26"E [±500 m], 1100 m, 20–21 December 1988, G. Monteith and G. Thompson, ex QM S18018, QM S91641.

##### Paratypes.

1 female, 1 stadium 7 male, 1 stadium 5 male, details as for holotype but 20 December 1988, berlesate 825, rainforest, sieved litter, QM S91640; 1 male, 1 female, 7 km N of Mt Spurgeon, Qld, camp 2, 16°22'31"S, 145°12'49"E [±500 m], 1200 m, 17–19 October 1991, G. Monteith, H. Janetzki, D. Cook and L. Roberts, QM S91639.

##### Other material.

1 male, Alexandra Bay, Qld, 16°12'S, 145°26'E [±2 km], <50 m, 24 June 1971, R.W. Taylor and J. Feehan, berlesate 331, rainforest, ANIC 64–000211; 1 male, 1 female, 1 stadium 7 male, Bargoo Creek, Windsor Tableland, 35 km NNW of Mt Carbine, Qld, 16°14'51"S, 145°04'08"E [±500 m], 850 m, 18 April 1982, G. Monteith, D. Yeates and D. Cook, berlesate 397, rainforest, sieved litter, QM S91638; 1 male, 4.0 km W of Mt Tribulation, Qld, site 8, 16°04'44"S, 145°25'59"E [±500 m], 720 m, 2 January 1983, G. Monteith, berlesate 503, rainforest, sieved litter, QM S91636; 1 male, 4.5 km W of Cape Tribulation, Qld, site 9, 16°04'41"S, 145°25'46"E [±500 m], 760 m, January 1983, G. Monteith and D. Yeates, berlesate 531, rainforest, sieved litter, QM S91634; 2 males, 4 females, same details but G. Monteith, berlesate 515, QM S91635; 1 stadium 7 female, 2.5 km N of Mt Lewis via Julatten, Qld, 16°33'49"S, 145°15'51"E [±500 m], 1040 m, D. Yeates and G. Thompson, berlesate 611, rainforest, sieved litter, QM S91642; 1 male, 2 females, North Bell Peak, Qld, 17°05'06"S, 145°52'00"E [±500 m], 600 m, 22 November 1990, G. Monteith and G. Thompson, berlesate 845, rainforest, sieved litter, QM S91643; 1 male and 1 female in copula, 1 stadium 7 male, 1 stadium 7 female, Roaring Meg valley, Qld, 16°03'45"S, 145°25'06"E [±500 m], 720 m, 22 November 1993, G. Monteith, H. Janetzki, L. Roberts and D. Cook, QM S91633; 2 males, 3 females, 1 stadium 7 male, Mt Halcyon, Qld, 16°03'16"S, 145°25'16"E [±500 m], 870 m, 22–24 November 1993, G. Monteith, H. Janetzki, D. Cook and L. Roberts, QM S91632; 2 males, 2 females, 1 stadium 7 male, Mt Hemmant, Qld, 16°06'44"S, 145°24'58"E [±500 m], 1050 m, 27 November 1993, G. Monteith and H. Janetzki, berlesate 865, rainforest, sieved litter, QM S91637.

**Figure 4.**
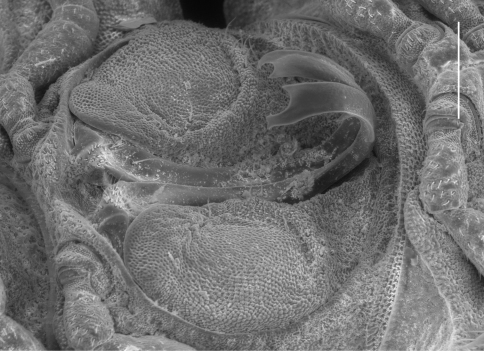
Left ventrolateral view of gonopods of *Prosopodesmus monteithi* sp. n., QM S91632. Scale bar = 0.25 mm. Image contrast is low because specimen is uncoated.

**Figure 5.**
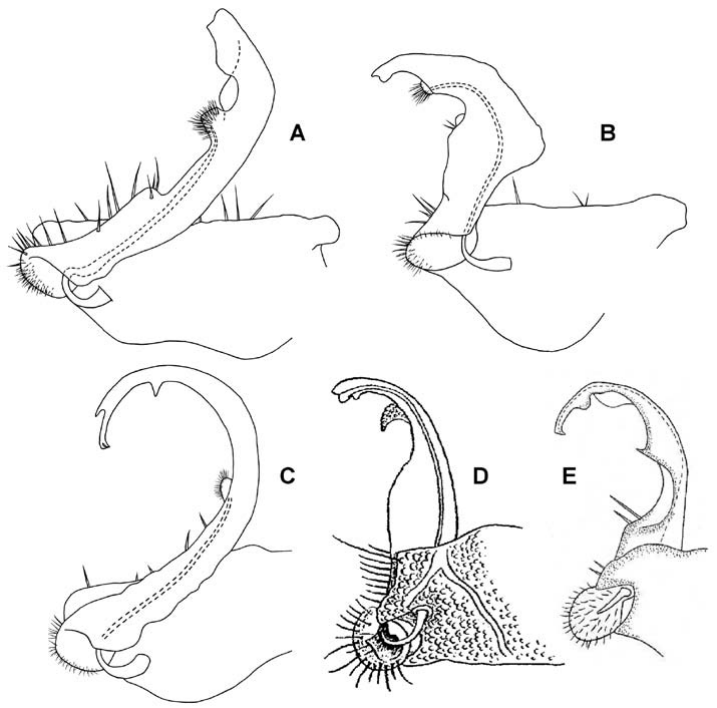
Medial views of right gonopod of *Prosopodesmus* species, not to same scale. **A** *Prosopodesmus crater* sp. n., paratype, ANIC 64–000214. **B** *Prosopodesmus kirrama* sp. n., paratype, QM S91626. **C** *Prosopodesmus monteithi* sp. n., QM 91635. **D** *Prosopodesmus sinuatus* (Miyosi, 1958), holotype, drawing scanned and modified from Fig. 1G in Miyosi (1958). **E** *Prosopodesmus similis* (Haga, 1968), holotype, drawing scanned and modified from Fig. 12B in Haga (1968).

**Figure 6.**
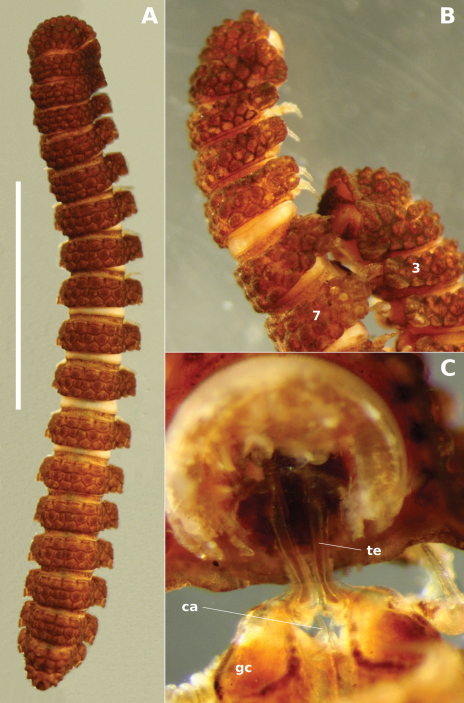
*Prosopodesmus monteithi* sp. n. **A** Adult female ex QM S91632; scale bar = 5 mm. **B** Mating pair ex QM S91633 with male ring 7 and female ring 3 labelled. **C** Partial dissection of the mating pair in **B** **ca** cannula, **gc** gonocoxa, **te** telopodite.

##### Diagnosis.

Males and females with head + 20 rings; adults 14–15 mm long; midbody metatergites typically with 3 transverse rows of 10 large tubercles; posterior portion of prozonite microvillose, without small disks or microtubercles; ozopores not on porosteles; gonopod telopodite slender, curved smoothly in J-shape.

##### Description.

As for *Prosopodesmus crater*, differing in the following details:

Male/female lengths ca 14/15 mm, respectively; adults light to medium brown (Fig. 6A, 6B). Ring 12 with maximum vertical diameter 1.2 mm; maximum width (including paranota) 2.0 mm and 1.8X prozonite width. Antennomere relative widths 5>6>(2,3,4), relative lengths (2,6)>(3,4,5). Collum, tergite and metatergite tubercles polygonal, closely fitted. Posterior portion of prozonite (Figs 2C, 2D) irregularly rugose and finely microvillose, without disks or microtubercles. Posterior notch on paranota at ca 2/3 paranotal length, anterior notch sometimes indistinct; paranota declined at ca 30°.

Telopodite (Figs 4, 5C) slender, smoothly curving in J-shape; hairpad mound at about midlength; a small triangular tab directed basally near curved-over tip; tip apically slightly excavate, the lateral side extended and terminating in 3 minute, finger-like processes.

##### Distribution.

Rainforest from Daintree National Park west of Cape Tribulation to the Malbon Thompson Range on the coast southeast from Cairns in Queensland, a north-south range of ca 125 km (Fig. 8).

##### Etymology.

For Geoff Monteith, former curator of insects at the Queensland Museum. Geoff and his colleagues collected most of the specimens of the three new *Prosopodesmus* species described in this paper.

##### Remarks.

*Prosopodesmus monteithi* is the largest known *Prosopodesmus* and the striking dorsal macrosculpture is easily visible to the unaided eye (Fig. 6A). One of the Queensland Museum samples contained a mating pair (Fig. 6B) which I partially dissected (Fig. 6C). As expected, the telopodites were rotated 90° out of the gonocoxal cavity in which they normally lie. The curved distal portion of each telopodite (Fig. 5C) was fully inserted into the cavity anterior to the epigyne, but how much of the curve was actually in contact with the cyphopod could not be seen, and would be better investigated with fixed, sectioned material.

#### Prosopodesmus panporus

Blower & Rundle, 1980

http://species-id.net/wiki/Prosopodesmus_panporus

Figs 2E 7

Prosopodesmus panporus Blower and Rundle, 1980: 27; figs 1-3, 6-8; table 1. Golovatch et al. 2009: 3.

##### Holotype.

Male, Palm House, Royal Botanic Gardens, Kew, London, UK, 16 May 1976, A.J. Rundle, extracted by hand from leaf litter in bed No. 1, slide-mounted in balsam, NHM. (Not examined.)

##### Paratypes.

1 male, details as for holotype, VMNH; 2 males, details as for holotype, IEA; 1 male and 1 female found in copula, details as for holotype but 15 April 1976, bed No. 2, NHM; 75 males, 41 females, 204 juveniles, details as for holotype but including Tullgren-extracted specimens, NHM. (Not examined.)

[Type details from Blower and Rundle (1980).]

##### Material examined.

9 males, 4 females, 9 km ENE of Mt Tozer, Qld, 12°43'S, 143°17'E [±2 km], 5–10 July 1986, T. Weir, ANIC berlesate 1057, rainforest litter, ANIC 64-000118; 15 males, 1 female, same details but ANIC berlesate 1059, ANIC 64-000210.

**Figure 7.**
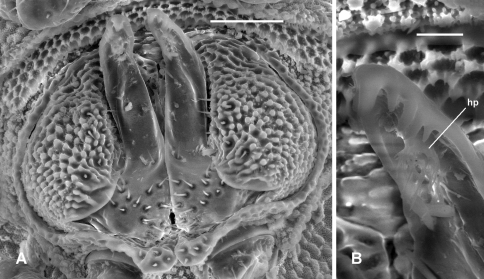
*Prosopodesmus panporus* Blower and Rundle, 1980, male ex ANIC 64–000210. **A** Gonopods in situ, ventral view **B** Close-up of left gonopod tip, showing hairpad **hp**. Image contrast is low because specimen is uncoated. Scale bars: **A** = 0.05 mm, **B** = 0.01 mm.

##### Diagnosis.

Males with head + 19 rings, females with head + 20; adults ca 3.5-4 mm long; midbody metatergites typically with 3 transverse rows of 10-12 large tubercles; posterior portion of prozonite with small disks and microtubercles; ozopores on porosteles on all podous rings beginning with ring 5; gonopod telopodite bent posteriorly at midlength, lateral edge near tip with several rounded teeth.

##### Description.

The excellent description and illustrations of Blower and Rundle (1980) are reproduced below in Appendix 1. Here I add a few details:

Antennal sockets separated by ca 1X a socket diameter. Antennomere relative widths (5,6)>(2,3,4); relative lengths 6>5>(2,3,4). Head with vertex and frons microtuberculate; clypeus smooth, sparsely setose dorsally. Dorsal and lateral setae on collum, tergites and metatergites sparse, bisegmented, the tips flared and minutely toothed along distal edge. Anterior portion of prozonite with cellular structure, the cell walls with microvillose extensions (Fig. 2E; see also Fig. 3 of Blower and Rundle (1980) in Appendix 1); posterior portion of prozonite more or less uniformly microtuberculate, raised into low mounds each topped with a more or less round, convex disk 3-4X the diameter of a microtubercle. Limbus a thin, straight-edged lamella (Fig. 2E). Sternites as wide as long. Podomere relative lengths femur>(prefemur, femur)>(postfemur, tibia). 5+5 lobes along posterior edge of telson in male examined with SEM (including bilobed epiproct), rather than 6+6 as noted by Blower and Rundle (1980). Spinnerets (the *four prominent setae...housed in a collar at the apex of the telson* of Blower and Rundle (1980, p. 29)) recessed in individual chambers, basal sheaths with unnotched distal edges.

Gonopore midventral on leg 2 coxa, opening on low, truncate conical process. Prostatic groove ending in hairpad in posterior concavity of telopodite (Fig. 7).

##### Distribution.

**Figure 8.**
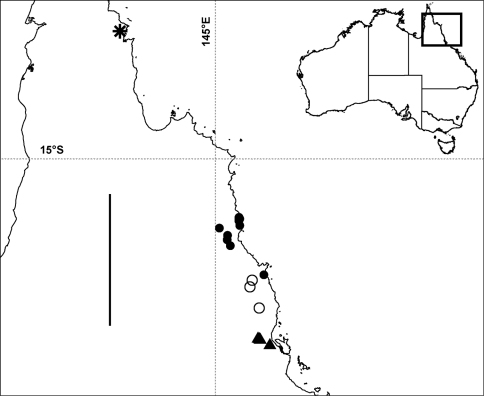
Localities in tropical north Queensland for *Prosopodesmus panporus* Blower and Rundle, 1980 (star), *Prosopodesmus monteithi* sp. n. (filled circles), *Prosopodesmus crater* sp. n. (open circles) and *Prosopodesmus kirrama* sp. n. (triangles). The questioned locality for *Prosopodesmus crater* (see text) is ca 700 km to the south of the furthest south *Prosopodesmus kirrama* locality and is not shown here. Geographic projection; scale bar = ca 250 km. Inset map of Australia shows location of main map.

So far known in Queensland from a single site in rainforest in the Iron Range Resource Reserve, ca 10 km northwest of Lockhart on the Cape York Peninsula (Fig. 8).

## Discussion

### Is *Prosopodesmus panporus* native to Queensland?

The Queensland *Prosopodesmus panporus* site is several kilometres inside a block of tropical rainforest several hundred square kilometres in extent. What is now the Old Coen Track, off Portland Roads Road, was used for vehicle access (Tom Weir, in litt., 24 April 2012). The site’s remoteness is evidence that *Prosopodesmus panporus* is native there rather than introduced. A second reason to think that *Prosopodesmus panporus* is native to Queensland is that three of its congeners, described in this paper, were collected in little-disturbed tropical rainforest ca 450 km to the south.

The case for native status would be even stronger if the Kew hothouses where *Prosopodesmus panporus* was first collected in 1975-76 were known to have contained plants imported from the Cape York Peninsula. I queried the Gardens but was told that glasshouse records for that period were not detailed enough to answer this question (Roxana Glenn, in litt., 11 April 2012).

It seems very likely, but not certain, that *Prosopodesmus panporus* is native to the Cape York Peninsula. Sequencing of a marker such as mitochondrial COI, from the Kew population and from specimens collected across the range of the species in Queensland, might help to locate the source or sources of the hothouse millipedes in the wild.

### Notes on morphology

The close resemblance of *Rhipidopeltis sinuata* to the *Prosopodesmus* species known at the time led Golovatch et al. (2009) to synonymise the two genera. They were aware that Miyosi (1958) had shown the prostatic groove terminating at the tip of the telopodite (Fig. 5D), rather than at a subapical hairpad. Golovatch et al. (2009) considered this difference to be only species-specific. Haga (1968) illustrated the gonopod of *Rhipidopeltis similis* with the prostatic groove ending, again, at the telopodite tip (Fig. 5E), and with no hairpad or other projection, only a tooth-like tab at midlength on the telopodite’s medial edge. The two species groups (with and without a hairpad termination for the prostatic groove) are clearly very closely related and I leave them here in *Prosopodesmus*.

The fine structure of the prozonite of *Prosopodesmus panporus* (Fig. 2E) corresponds exactly to that of *Prosopodesmus jacobsoni* as illustrated in Akkari and Enghoff (2011; Fig. 4, p. 7): anteriorly with cellular chambers whose walls are extended with microvilli, posteriorly with slightly elevated convex disks (‘subspherical knobs’ of Akkari and Enghoff) on a ground covered with microtubercles. These are not genus-level character states, since the other three Australian *Prosopodesmus* species lack microvillose extensions anteriorly and microtubercles posteriorly, and the posterior prozonite in *Prosopodesmus monteithi* lacks disks and is irregularly rugose with microvilli (Figs 2A–D).

I could not find spiracular openings above the legbases in any of the Australian *Prosopodesmus* species. Although this is consistent with the apparent lack of spiracles in two other Australian haplodesmids, *Agathodesmus johnsi* Mesibov, 2009 and *Asphalidesmus steeli* Silvestri, 1910 (Mesibov 2009), it is very puzzling. Spiracles are clearly visible in all known species of *Asphalidesmus* Silvestri, 1910 (Dalodesmidea) and in the Australian Pyrgodesmidae I have examined. Both groups are similar to *Prosopodesmus* species in body form, size range and cryptic habits in rainforest. Unless the two haplodesmid genera lack tracheae, which seems to me improbable, then their tracheal systems must have spiracular entrances. A histological study of freshly collected and suitably prepared *Prosopodesmus* specimens, e.g. the large *Prosopodesmus monteithi*, might solve this puzzle.

## Supplementary Material

XML Treatment for Prosopodesmus crater

XML Treatment for Prosopodesmus kirrama

XML Treatment for Prosopodesmus monteithi

XML Treatment for Prosopodesmus panporus

## Figures and Tables

**Table 1.** Comparison of *Prosopodesmus panporus* sp. n. with *Prosopodesmus jacobsoni* Silvestri.

*Prosopodesmus jacobsoni*	*Prosopodesmus panporus*
1. Males and females with 20 segments	Males with 19, females with 20 segments
2. Red-brown, 6.0 × 0.71 mm (nominate form)<br/> Yellowish, 7.7 × 0.95 mm (subsp. hilaris Brolemann)	Cream-white, males 3.3–3.8 mm × 0.45–0.50 mm<br/> Females 3.8–4.3 × 0.50–0.51 mm
3. Ozopores on segments 5, 7-–19	Ozopores on segments 5–17(18) males, 5–18(19) females
4. First row of metazonal tubercles largest, third row smallest (a fourth row in P. j. hilaris)	Tubercles of all three rows of equal size (posterior edge of metazonite could be regarded as a fourth row of flatter tubercles).
5. Posterior edge of telson with ten lobes	Posterior edge of telson with 12 lobes.
6. Gonopods with a large lateral lamella on proximal half of telopodite. Two subapical teeth (Figs. 4 & 5).	No lateral lamella on telopodite. Four (sometimes a fifth) subapical teeth (Figs. 6, 7, 8).

**Figure 1.**
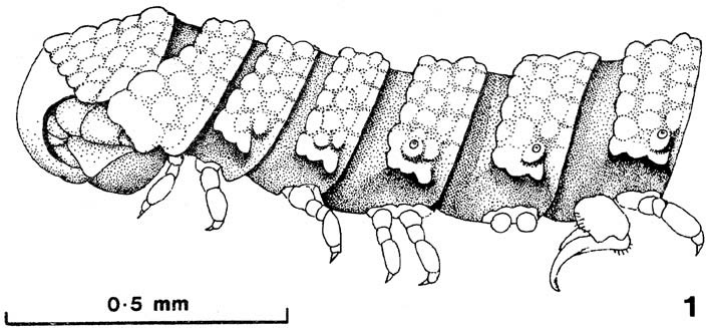
*Prosopodesmus panporus*, n. sp. Lateral view of the anterior part of the male of a mating pair collected in the Palm House, 15 April 1976 (paratype 5). [Reproduced with permission from Blower and Rundle (1980), p. 28]

**Figures 2–3.**
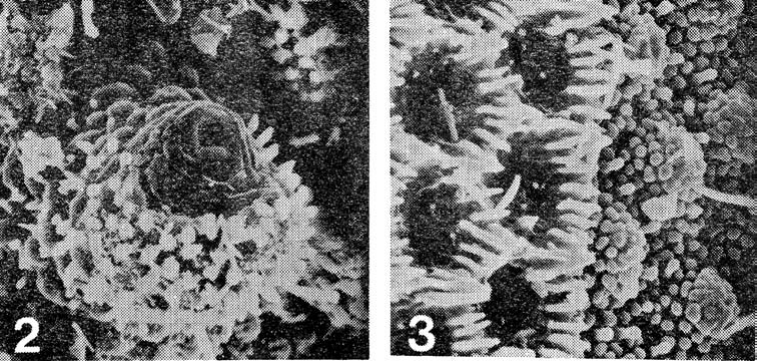
**2** *Prosopodesmus panporus*, n. sp. Electron scan photograph of the porostele and ozopore on the last podous segments of a female, X 775 **3** Detail of the junction of prozonum and metazonum of the 14^th^ ring of the same female, prozonum on the right, X 1,500. [Reproduced with permission from Blower and Rundle (1980), p. 29]

**Figures 4–8.**
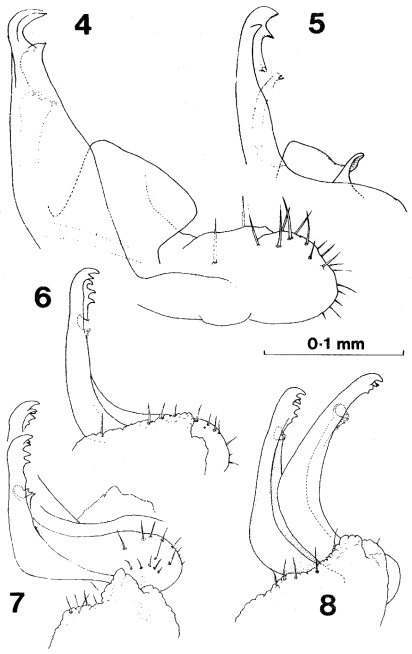
Gonopods of *Prosopodesmus* **4** *Prosopodesmus jacobsoni* Silvestri, right gonopod in external profile, drawn from holotype **5** Right gonopod, redrawn from 7 of the original description (Silvestri, 1910) **6** *Prosopodesmus panporus* n. sp. External profile view of right gonopod of paratype 2 (the specimen is mounted with left side uppermost, but the drawing is reversed to compare more easily with the other two) **7** *Prosopodesmus panporus*, gonopods of the holotype *in situ*, viewed from the right and slightly ventrally **8** *Prosopodesmus panporus*, gonopods of paratype 1 *in situ*, viewed from the right. [Reproduced with permission from Blower and Rundle (1980), p. 31]

## Appendix 1

Original description and illustrations of *Prosopodesmus panporus*, from Blower and Rundle (1980), pp. 27–31. [Reproduced with permission]

***Prosopodesmus panporus* sp. n.**

The following is a composite description based on all the material in our possession. Specific reference to one or another of the types is made in the legends to the figures.

Body almost pigmentless, white to cream-white. Males with 19 segments, 3.3–3.8 mm long, 0.45–0.50 mm broad; females with 20 segments, 3.8–4.3 mm long, 0.50–0.51 mm broad.

Head almost completely covered by a fan shaped (sectorial) collum; metazonite of second ring broader than the metazonites of all subsequent rings, the paranota forwardly directed, embracing the head and collum, their lateral edges confluent with the sides of the collum and continuing the lines of same; the division between the collum and second ring in life is not immediately evident in dorsal view.

The antennae are inserted close together on the vertex; when at rest, they occupy deep grooves diverging dorsally and then passing posteriorly, their distal antennomeres overhung by the sides of the collum and the paranota of the second ring. The forehead triangle enclosed within the diverging antennal bases and the anterior edge of the collum is raised into seven tubercles, one at the apex, two in the next row and four forming the base beneath the collum edge. The sixth antennomere is the longest and broadest; the fifth is slightly shorter. Each of these two larger antennomeres carries a group of sensory cones on their distal dorsal extremities.

The dorsal surface of the collum is raised into an even pavement of tubercles; those of the outermost row are flatter than the remainder and form the incised anterior edge of twelve lobes; these are followed by four rows of more convex tubercles. The succeeding metazonites are strongly arched and are raised into three transverse rows of regular tubercles uniform with those of the collum. The metazonites of the third and fourth rings are much less broad than those of the second ring and slightly less broad than the fifth and all subsequent rings except the telson. The paranota are placed slightly below the mid-lateral level and are incised into three lobes; the middle lobe is slightly broader but not more protrusive; the posterior lobe is more clearly separated from the other two and is directed slightly postero-laterally.

Ozopores occur on all diplopodous segments, 5–17 in the male and 5–18 in the female and there is a pair of rudimentary pores on the single apodous ring (ring 18 of the male and 19 of the female). The pores are borne on the outermost pair of tubercles, of the middle row of the fifth and of the posterior row of the sixth to the last podous ring. The poriferous tubercles of the fifth metazonite obscure the middle lobe of the paranotal edge in dorsal view; those of the remaining diplopodous rings obscure the posterior paranotal lobes. The poriferous tubercles are directed slightly posteriorly, a tendency most obviously developed on the last two or three diplopodous rings where they project posterolaterally beyond the posterior limit of the metazonite.

The anterior row of tubercles on the metazonites slightly overhang the anterior edge. The third row of tubercles stop just short of the posterior edge which is incised into flat fields corresponding in number to the tubercles; these flat posterior portions of the metazonite could just conceivably be described as a fourth row of rather flat tubercles. There are eight tubercles in the first and second rows of the anterior rings, passing to ten on the sixth and eventually twelve from the eighth onwards. The posterior row on rings one to five include 9 tubercles increasing to 11 from the sixth to the last ring, the outermost pair carrying the ozopores, which on the fifth ring are borne on the outermost pair of tubercles of the second row. The telson carries the same three rows of tubercles although the numbers in the second and third rows naturally diminish towards the apex. The tubercles along the posterior edge of the telson overhang as 6+6 lobes.

The entire surface of the tubercles and the region between them is raised into microtubercles and micropapillae, as also is the surface of the prozonites (figs 2, 3). The only macrosetae are carried on the telson, anal valves and scale; there are 4+4 inserted just beneath the twelve-lobed posterior edge of the telson, level with the dorsalmost 3+3 of these lobes. A further four prominent setae are housed in a collar at the apex of the telson just below the medianmost lobes. Each of the anal valves carries 2+2 setae and a further pair are carried by the trapezoidal anal scale. None of these macrosetae is visible from the dorsal side due to the ventrally directed curvature of the telson.

The gonopods are shown in figs 6–8 along with those of *Prosopodesmus jacobsoni*. In *Prosopodesmus panporus* they are simple uniramous structures terminating in prominent teeth, one apical, two sub-apical arising at about the same level and a further two (sometimes a third rudimentary tooth) along the external edge of the ramus just below the first sub-apical pair. Figs 6–8 indicate different appearances of these teeth when viewed from different aspects.

## Appendix 2

Records of species of *Prosopodesmus* Silvestri, 1910 in Australia.

**Explanation note:** Specimen records for *Prosopodesmus* species in Australia are available on the ZooKeys website as a CSV file (doi: 10.3897/zookeys.190.3276.app2).
